# Transferring intercellular signals and traits between cancer cells: extracellular vesicles as “homing pigeons”

**DOI:** 10.1186/s12964-016-0136-z

**Published:** 2016-06-10

**Authors:** Giulia Cesi, Geoffroy Walbrecq, Christiane Margue, Stephanie Kreis

**Affiliations:** Life Sciences Research Unit, University of Luxembourg, 6, av. du Swing, L-4367 Belvaux, Luxembourg

**Keywords:** Extracellular vesicles, Intercellular communication, Imaging, Cancer, Drug resistance

## Abstract

Extracellular vesicles are cell-derived vesicles, which can transport various cargos out of cells. From their cell of origin, the content molecules (proteins, non-coding RNAs including miRNAs, DNA and others) can be delivered to neighboring or distant cells and as such extracellular vesicles can be regarded as vehicles of intercellular communication or “homing pigeons”. Extracellular vesicle shuttling is able to actively modulate the tumor microenvironment and can partake in tumor dissemination. In various diseases, including cancer, levels of extracellular vesicle secretion are altered resulting in different amounts and/or profiles of detectable vesicular cargo molecules and these distinct content profiles are currently being evaluated as biomarkers. Apart from their potential as blood-derived containers of specific biomarkers, the transfer of extracellular vesicles to surrounding cells also appears to be involved in the propagation of phenotypic traits. These interesting properties have put extracellular vesicles into the focus of many recent studies.

Here we review findings on the involvement of extracellular vesicles in transferring traits of cancer cells to their surroundings and briefly discuss new data on oncosomes, a larger type of vesicle. A pressing issue in cancer treatment is rapidly evolving resistance to many initially efficient drug therapies. Studies investigating the role of extracellular vesicles in this phenomenon together with a summary of the technical challenges that this field is still facing, are also presented. Finally, emerging areas of research such as the analysis of the lipid composition on extracellular vesicles and cutting-edge techniques to visualise the trafficking of extracellular vesicles are discussed.

## Background

**Fig. 1:**
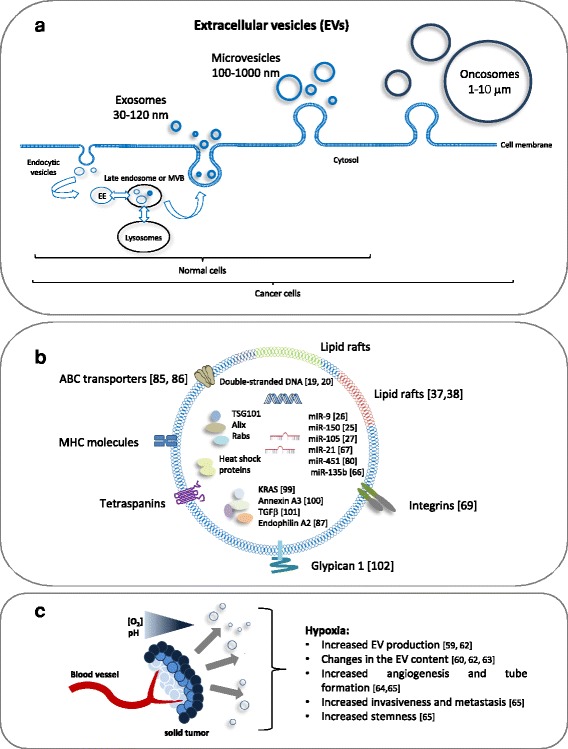
**a** EV repertoire. Cells secrete distinct sub-populations of EVs and although of different origins, they overlap in size or density and often co-purify. Exosomes are vesicles generated by the inward budding of the endosomal compartments (endocytic vesicles), which become early endosomes (EE), several of which are forming so-called multivesicular bodies (MVBs). MVBs either fuse with lysosomes or with the plasma membrane, which results in their secretion. In addition, cancer cells can produce larger vesicles named “large oncosomes”. Together with exosomes and microvesicles, oncosomes contain abundant bioactive molecules, which can transfer cancer traits or be used as biomarkers. **b** Relative to the general EV content of normal cells (tetraspanins, MHC molecules, proteins involved in the MVB biogenesis, heat shock proteins), cancer EVs are often enriched in specific miRNAs or proteins. Furthermore, the membrane of cancer EVs is characterised by specific lipid species localised in lipid rafts. **c** Cancer cells secrete more EVs than the corresponding healthy cells. Acidic pH and hypoxia, which often characterise the tumor microenvironment, stimulate an increased secretion of EVs and influence the EV content, which in turn supports angiogenesis and metastasis. Additional references not previsouly cited in the text: KRAS [99], Annexin A3 [100], TGFβ [101], Glypican 1 [102]

According to an advanced PubMed search, “exosome” has become the most cited term in publications describing any kind of vesicle [1]. However, the term exosome only refers to vesicles generated by the inward budding of the endosomal compartments, of which several are forming so-called multivesicular bodies (MVBs). Many MVBs fuse with lysosomes whereas others may fuse with the plasma membrane, resulting in secretion of their intraluminal vesicles [2]. Exosomes are generally distinguished from microvesicles by size and by origin: exosomes are ~30 -120 nm in size, with an endosomal origin whereas microvesicles are >100-1000 nm and originate from the plasma membrane (Fig. 1a). Current purification methods unfortunately do not allow to precisely discriminate between the two populations as it is very likely that microvesicles with a size of 30–120 nm exist as well. Protein aggregates and lipoproteins might also contaminate and confound the sample preparation. Furthermore, once the vesicles have been released, their origin cannot be identified as unique markers for the different vesicle types have not been defined yet [3–5]. Because of the difficulty to specifically discriminate exosomes from other circulating vesicles, the International Society of Extracellular Vesicles suggested to use the generic term “extracellular vesicles (EVs)” to describe vesicles isolated from the extracellular milieu [1]. For clarity, in this review, the term exosome mentioned in the majority of the cited publications, will be replaced by the term “extracellular vesicles” to be in accordance with the new guidelines.

Although various isolation methods exist, differential ultracentrifugation followed by density gradient separation, electron microscopy together with protein composition analysis is generally considered the best so far available workflow to isolate and characterise EVs [6]. Nevertheless, some studies where EVs were isolated by using the less accepted “precipitation method” are also cited here, in order to provide a complete overview of the different topics covered in this review.

EVs were initially thought to be an expedient for cells to remove unwanted components [7, 8]. However, recent findings have shown that these nano-vesicles are surely more than just garbage bins. As EVs can be released by “donor” cells and taken up by “recipient” cells, it has been hypothesised that these vesicles broaden the cells’ repertoire to communicate and exchange signals. Several studies have already confirmed that EVs are important players in influencing both physiological and pathological conditions by delivering molecules such as cytokines, growth factors, proteins, mRNAs, miRNAs and other non-coding RNAs to other tissues and cells [3]. The discovery that EVs have a wide range of regulatory functions and carry various endogenous cellular components made them the most studied vesicles in recent years [9] and several reviews covering different aspects of extracellular vesicle biology and function have been published [9–13].

The current review focuses on new developments in EV characterisation (lipid profiling of cancer-derived EVs) and visualisation (imaging of EV traffic) as well as on the potential involvement of EVs in propagating tumorigenic properties, in particular drug resistance. Finally, technical limitations that impede a full understanding of EV biology and functions will be summarised.

## Characterisation of EVs

### Cargo and composition of EVs

The content profiles of EVs depend mainly on their parental cells. The ExoCarta database (www.exocarta.org) provides information about the EV content in different organisms and cell types. Generally, EVs from different cell types contain endosome-associated **proteins** (e.g. Rab GTPase, SNAREs, Annexins, and flotillins), some of which are involved in the biogenesis of MVBs (e.g. Alix and Tsg101) [14]. Membrane proteins including tetraspanins (e.g. CD63, CD81, CD82, CD53, CD37 and CD9), heat shock proteins, MHC complexes, growth factors and many others are also present [15]. How exactly proteins are sorted into EVs is still under investigation. The role of ubiquitination seems controversial: in most cases, ubiquitination targets the proteins destined for degradation (upon fusion of the MVB with lysosomes) while proteins to be exported show no ubiquitination [16]. In some other cases, EV proteins appear to be highly ubiquitinated [17]. EVs have also been shown to contain single-stranded **DNA** and transposable elements [18] as well as double-stranded genomic DNA which might reflect the mutational status of the parental tumor cell [19, 20].

The **RNA** content of EVs is enriched in small non-coding RNAs including **miRNAs** [2]. Although the sorting mechanisms are not fully understood, recent evidence suggests that the composition of EV miRNAs differs from the one of the cell of origin suggesting a selective sorting of miRNAs into EVs. In this context, Villarroya-Beltri et al. [21] identified the presence of unique sequence motifs that could prone miRNAs for sorting into EVs or for intracellular localisation. They also demonstrated that the sumoylated heterogeneous nuclear ribonucleoprotein A2B1 binds miRNAs through their “EXO-motifs” and controls their loading into EVs, thus providing an explanation for the specific packing of certain miRNAs into EVs. In contrast, interesting findings from Squadrito et al. [22] suggest a passive mechanism for miRNA export modulated by cell activation-dependent changes of miRNA target levels: EV miRNA secretion might be a mechanism by which cells remove miRNAs in excess of their corresponding targets to re-establish miRNA/mRNA homeostasis. More recently McKenzie and collegues identified Ago2 protein as a possible major player in miRNA sorting. Indeed, they demonstrated that phosphorylation of Ago2 promoted by KRAS suppressed its secretion into EVs and thereby the sorting of specific miRNAs [23].

The presence of regulatory miRNAs within EVs has raised a strong interest ever since Valadi et al. [24] showed for the first time that miRNAs in mast cell-derived EVs can be transferred to other mast cells and be functional. Since then, fascinating examples of intercellular communication via miRNAs between cells in culture have been provided [25–28]. Albeit accumulating evidence for the importance of miRNAs in EVs, it remains uncertain whether such miRNAs are really functional in a physiological environment and whether the concentration of secreted individual miRNAs would be sufficient to mediate measurable endocrine effects. Furthermore, it is still unclear how widely this process occurs *in vivo* and whether it is restricted to certain cell types, physiological conditions or diseases or whether it is a ubiquitous way of cell-to-cell communication. For Williams et al. [29] the concentration of miRNAs in biological fluids is significantly lower than in the surrounding cells and might be below the threshold for triggering any significant function *in vivo*. The work of Chevillet et al. [30] argues along these lines. By using a stochiometric approach, they performed quantitative assessments of miRNAs within EVs isolated from five different sources. Less than one copy of a given miRNA per EV was observed by absolute quantification through real time PCR. These data would suggest that standard EV preparations might not carry biologically significant numbers of miRNAs. In accordance with this, we made a similar observation. After successful transfer of detectable levels of miR-211-5p via EVs isolated from melanoma patient serum samples to miR-211-5p-negative melanoma recipient cells, we could not detect any down-regulation of previously confirmed target genes (unpublished data). Interestingly, in the same cellular model 5 nM of miR-211 mimic was able to effectively down-regulate those target genes (RAB22A, AP1S2, M6PR) [31] suggesting that amounts of transferred miRNAs isolated from patient sera were not sufficient to evoke downstream effects. Moreover and apart from quantities, other factors play a role: it is still difficult to clearly discriminate between secreted miRNAs indicative of malignant processes from “contaminating” miRNAs derived from platelets, erythrocytes, lymphocytes or normal cell death [32]. Among all the components of EVs, miRNAs are one of the most controversial but also interesting players in intercellular signaling and tumor progression and their potential involvement in acquisition and transfer of cancer cell resistance to drug treatments is discussed in more detail below.

EVs carry **lipids** of a similar composition as found in the plasma membrane of the parental cells (such as cholesterol, ceramide and sphingomyelin) [2]. An emerging field in vesicle research and more specifically in the context of cancer, is “**lipidomics**” which, apart from general lipid profiling, also studies alterations in lipid compositions. Changes in lipid metabolism and in particular activation of *de novo* lipogenesis have already been described for several cancers [33–35]. Recently, Marien and colleagues identified a distinct lipid signature in non-small cell lung cancer. By using a mass spectrometry-based phospho-lipidomics approach, the authors identified 91 phospholipid species differentially expressed in cancer versus normal tissues [36]. The distinct lipid composition of EVs coupled with the capability of EVs to travel in biological fluids, puts lipid profiling on the list for novel biomarker discovery. Interestingly, an enrichment in certain lipid species in the membrane of EVs has been reported in several publications. In this context, Llorente et al. [37] observed a specific sorting of lipids into EVs compared to the secreting cells. Lipid composition analysis of metastatic prostate cancer cells and corresponding EVs revealed an enrichment in glycosphingolipids, cholesterol, sphingomyelin and phosphatidylserine in EVs compared to parental cells. However, the authors did not compare the lipid composition of these EVs to those released from normal prostate cells. The enrichment of specific lipids within the membrane of EVs has also been described in colorectal cancer cells [38]. Furthermore, Schlaepfer and colleagues observed that hypoxia triggered triglyceride accumulation in prostate cancer cells and corresponding EVs due to the activation of lipogenesis-related enzymes [39]. Overall, lipidomics of EVs has gained attention in recent years but to this day, it remains controversial which lipids are involved in EV-mediated cell-to-cell communication [40], also because it is a challenge to produce pure EV preparations and to avoid cellular lipoparticle contaminations, potentially leading to misinterpretations. Nevertheless, standardised and well-controlled lipid profiling of EV membranes might be useful for the identification of new biomarkers and for a better understanding of the biology of EV secretion.

### Visualisation of EVs and EV traffic

The most common methods used to detect and characterise EVs are electron microscopy (EM), dynamic light scattering (DLS), nanoparticle tracking analysis (NTA), fluorescence microscopy and flow cytometry (FCM). Two standard methods are used to assess the quality of the EV preparation: EM and either DLS or NTA. EM has the advantage that it provides the highest resolution compared to the other methods. In addition, EM combined with immuno-gold labeling allows for recognition of protein markers on the surface of EVs. DLS and NTA both measure the size of particles using Brownian molecular movement but NTA has, additionally, a camera documenting the movement and light scattering of the samples [41]. Unlike previous methods, which only enable physical characterisation of EVs in fixed samples, fluorescence microscopy visualises labelled EVs in live cell conditions/assays. Several fluorescent membrane dyes are used to label purified EVs such as the PKH-67 (green) or PKH-26 (red) linker dyes. One disadvantage of the labelling dyes is their long half-life *in vivo* (from 5 to >100 days), which hinders the dynamic tracking of EVs *in vivo* [42].

An alternative to labelling the membranes of EVs is to link their protein content to TAMRA-NHS (carboxytetramethylrhodamine succinimidyl ester, Biotum) [43]. In order to label EVs released by the cells *in vitro* and *in vivo*, EV protein marker or membrane localisation tags (e.g. palmitoylation signal) have been fused to fluorescent proteins [44, 45]. Moreover, EVs from melanoma cells were visualised *in vivo* using multiphoton microscopy in orthotopic tumors using *Gaussia* luciferase (Gluc) [46]. Gluc was also fused to biotin (GlucB) on the surface of the EVs, facilitating the conjugation of labeled streptavidin in order to see the labeled EVs *in vivo* using fluorescence mediated tomography (FMT) [42]. In addition to FMT, GlucB can also be visualised using magnetic resonance imaging (MRI) or positron emission photography (PET) [47]. Finally, advances in flow cytometry (FCM) enhanced the sensitivity of this technique to detect EVs. A recent, improved method allows for detection of PKH-67-labelled EVs with a comparable detection threshold as compared to NTA [48]. FCM can also be coupled with a camera in order to discriminate the EVs from false positive results [49, 50] and has been applied to characterise EVs released by mesenchymal stromal cells (MSC) using antibodies against MSC marker proteins [51].

Lai et al. have recently succeeded to show the dynamics of EV-mediated communication by taking advantage of the different combination possibilities offered by fusing enhanced green fluorescent protein (eGFP) and tandem dimer Tomato (td Tomato) to a palmitoylation signal (PalmGFP, PalmtdTomato) and GlucB [52]. First, EV exchange between 2 populations of cancer cells was visualised by labelling one with PalmGFP and the other with PalmtdTomato [52]. Then, by combining Gluc-labelled EV [42] and PalmtdTomato, EV uptake and EV-mRNA translation was tracked in the recipient cells [52]. Next, EV-packaged mRNA was monitored by tagging the transcripts encoding PalmtdTomato to a MS2 RNA binding sequence fused with eGFP, allowing to simultaneously visualise EV-packaged mRNA and EVs themselves. Finally, by combining PalmtdTomato and EV-GlucB, the dynamics of EV uptake and EV-mRNA translation were monitored [52]. Applying completely different systems to track EV traffic, several elegant studies by two different groups have visualised EV uptake in living cells using both β-galactosidase and the Cre/LoxP system. Ridder et al. used LacZ gene as reporter gene and β-galactosidase expression was induced in recipient cells by EV transfer. This transfer was demonstrated in mouse tumor models and between hematopoietic system and brain *in vivo* [53, 54]. This method was the first approach to analyse the physiological transfer of EVs *in vivo* and represents a step forward in avoiding potential artifacts introduced by submitting cells to an excess amount of isolated and labelled EVs [55]. By using a similar approach, the expression of green fluorescent protein (GFP) was triggered in cells, which took up EVs produced by tumor cells expressing the Cre recombinase [55, 56]: Cre-expressing melanoma cells injected into mice were releasing EVs containing Cre mRNA, which were then transferred to non-tumour cells *in vivo* [56]. In the target cells, Cre mRNA was translated into Cre protein and induced the expression of GFP [56]. However, this method cannot be used to assess the precise quantification of the uptake of EVs in recipient cells [56] as it does not allow for characterisation of transferred EVs or the uptake mechanism of EVs [57].

Taken together, the visualisation and tracking of EV movements has seen rapid and promising developments in recent years. Nevertheless, all these advances will need appropriate controls and to some degree standardisation of protocols in order to substantiate new findings on EV dynamics, characteristics and transfer of oncogenic traits in physiologic contexts and their potential clinical applications. As such, these novel imaging techniques could be combined with gene deletion or mutation strategies in order to better understand the role of specific molecules, which are transferred into EVs or are involved in the loading of cargo into EVs or in the uptake of EVs by target cells.

## EVs in cancer

Cancer EVs differ from those released by healthy cells in terms of content and quantity. An increased secretion of EVs has been reported for different cancer cell lines and patients [58, 59]. Some typical proteins, miRNAs and other molecules described to be augmented in EVs released from cancer cells are presented in Fig. 1b. Acidic pH [60, 61] and **hypoxia** [59, 62], hallmark properties of many solid tumors, might be responsible for the intensification of EV production and for their altered content. Several recent studies have investigated this phenomenon under hypoxic conditions. An enhanced secretion of microvesicles from mesenchymal stem cells in response to hypoxia was reported by Zhang et al. [63]. Along these lines, Kucharzewska et al. [64] showed that EVs derived from glioblastoma cells grown under hypoxic conditions were potent inducers of angiogenesis *in vitro* through phenotypic modulation of endothelial cells: glioblastoma-derived hypoxic EVs induced endothelial cells to secrete several potent growth factors and cytokines and to stimulate the PI3K/AKT signaling pathway. In addition, EVs from hypoxic prostate cancer cells enhanced invasiveness and stemness of prostate cancer cells under normoxia and promoted the cancer-associated fibroblast phenotype in prostate stromal cells by targeting adherent junction molecules [65]. Umezu and colleagues [66] provided evidence that in endothelial cells, hypoxia-driven accelerated tube formation was attributable to miRNA-135b in EVs shed from hypoxia-resistant multiple myeloma cells. Interestingly, the EV transfer of miRNA-135b resulted in the suppression of FIH-1, a negative regulator of HIF-1α suggesting that the upregulation of HIF-1α could enhance angiogenesis. More recently, Li et al. observed increased levels of miR-21 in EVs isolated from hypoxic oral squamous cell carcinoma. The transfer of this miRNA in normoxic cells induced migration and invasion both *in vitro* and *in vivo* [67]. Although the mentioned studies of Umezu and Li provide solid evidence for the reported biological effects, they did not follow the generally accepted EV isolation procedures, increasing the possibility of precipitating contaminants.

It is also worth mentioning that hypoxic, but not normoxic tumor-derived EVs impaired NK cell function by delivering both TGFβ and miRNA-23a [68]. In conclusion, EVs secreted from hypoxic cancer cells seem to carry a cargo, which supports angiogenesis and thus metastasis as well as immunosuppression.

Cancer EVs are taken up by “recipient cells” but whether this process occurs in a specific manner or at random is poorly understood. Interesting findings by Hoshino et al. showed that integrins inserted in the membrane of EVs dictate their adhesion to specific cells in specific organs [69]. Tumor-derived EVs taken up by specific cells based on their integrin expression profile, were able to promote pro-migratory and pro-inflammatory S100 gene upregulation and by doing so, initiated the pre-metastatic niche *in vivo*. More studies will be necessary to confirm a potential specificity in the cellular uptake of EVs.

EVs are not the only vehicles to transfer oncogenic information. Recently, a new class of microvesicles named **oncosomes** has been described. Oncosomes differ from nano-sized EVs in terms of size (oncosomes are much larger with a diameter of 1–10 μm) and in the biogenesis pathway (oncosomes derive from the plasma membrane of cells that have acquired an amoebotic phenotype, Fig. 1a). Di Vizio et al. [70] first introduced the term “large oncosomes” to describe large vesicles originating from amoeboid prostate cancer cells. The shedding of these vesicles could be induced by EGF while bleb formation in both normal prostate epithelial and stromal cells was modest and unresponsive to EGF. Indeed, only tumor cells seem to release quantifiable amounts of large oncosomes that were directly correlated with their rate of aggressiveness [71, 72]. Oncosomes, like EVs, contain mRNAs, miRNAs and proteins. Caveolin 1, a serum biomarker of metastatic prostate cancer, was detected in oncosomes and thus correlated with prostate tumor progression in mice and discriminated patients with metastatic disease from those with organ-confined disease [71]. Furthermore, the oncosome-associated miR-1227 produced by prostate cancer cells was able to induce migration of cancer-associated fibroblasts [72]. Whether nano-sized EVs and oncosomes share some of their molecular cargo is still under investigation. Nevertheless, recent findings from Minciacchi et al. revealed a different protein content in the two vesicle populations suggesting a specific selection of proteins destined for both vesicle types [73]. So far only a limited number of studies are available on oncosomes and more will be required in order to better characterise the two vesicle classes in terms of unique markers, content and function.

## Contribution of EVs to drug resistance

**Fig. 2:**
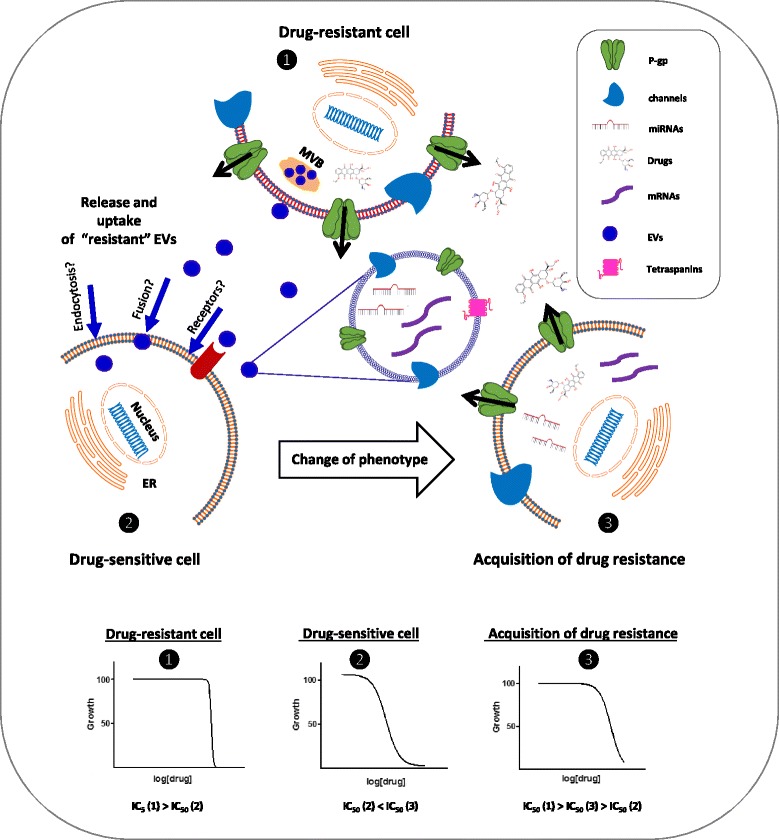
Drug-resistant cells can transfer the resistant phenotype through EVs. EVs released by drug-resistant cells contain proteins and miRNAs, which partake in propagating resistance. Drug-sensitive cells become gradually resistant when they incorporate ”resistant EVs”: Resistant cells (1) then over-express efflux pumps (P-gp) to eliminate anti-cancer drugs and produce more EVs, which again reflect the resistant phenotype of the secreting cell. Once in the extracellular environment, these EVs can be taken up by sensitive recipient cells (2) through fusion, endocytosis or binding to surface receptors. The released content acts on these cells, which in turn might also become drug-resistant (3). The lower part shows example trends of dose–response curves to a cytotoxic drug, representative of drug-resistant cells (1), drug-sensitive cells (2) and the same sensitive cells, which are becoming resistant after incubation with “resistant EVs” (3). The effectiveness of the drug to inhibit a specific biological function in the cells (exemplified by the inhibition of cell growth) is expressed by the IC_50_ value. The higher this value, the more resistant the cells. The IC_50_ value of cells which are acquiring the resistant phenotype would be in between the IC_50_ values of the other two conditions: IC_50_ (1) > IC_50_ (3) > IC_50_ (2)

Drug resistance of cancer cells represents a challenge in most anti-neoplastic treatments. The development of a resistant phenotype is considered to be multi-factorial and mainly due to decreased drug accumulation, increased efflux, increased biotransformation, drug compartmentalisation, acquired genetic modification of drug targets and/or defects in cellular pathways [74]. Recently, EVs have been identified as new players in passing resistance onto still sensitive cells [75, 76], which in turn might “gain” drug resistance traits as illustrated in Fig. 2.

### EV-mediated drug export

Apart from the up-regulation of efflux pumps, which will be described below, in the establishment of drug resistance, the direct sequestration of drugs into lysosomal vesicles and EVs has also been reported. Safaei et al. [77] have shown the lysosomal compartment to be notably reduced in size in cisplatin-resistant human ovarian carcinoma cells with more EVs exporting cisplatin via this route compared to sensitive cells.

Pulse-chase experiments with doxorubicin, a fluorescent anticancer drug, confirmed the hypothesis that drug expulsion can occur via EVs. In MCF-7 cells, doxorubicin localised in the nucleus immediately after the drug was administered. Twenty-four hours later, nuclear fluorescence was significantly decreased and most of the visible doxorubicin was present in EVs associated with the cell periphery indicating the active sorting of drugs into vesicles [78]. Furthermore, Federici et al. [61] demonstrated that EVs purified from supernatants of melanoma cells treated with cisplatin contained detectable levels of the drug. HPLC analysis indicated that cisplatin within EVs was in its unmetabolised form suggesting that EVs might incorporate the drug immediately after the uptake by the cell. More recently Koch et al. were able to detect anthracyclines in EVs from diffuse large B cell lymphoma cells lines. Interestingly, knocking down the ATP-transporter A3 (ABCA3) augmented intracellular retention of the drugs, thus increasing their cytostatic effects [79].

### EV-mediated miRNA export

In addition to the above-mentioned mechanisms, miRNAs packaged within EVs can also contribute to the onset and maintenance of drug resistance. Pigati et al. [80] have observed that mammary epithelial cells released a different subset of miRNAs compared to the ones which were retained. They found that nearly 30 % of the released miRNAs *in vitro* did not reflect the cellular profile, indicating that miRNAs are retained or released selectively. In particular, the malignant mammary epithelial cells released most of their miRNA-451 into the environment. This concurs with findings from Kovalchuk et al. [81] who reported that miRNA-451 targets the multidrug resistant gene (mdr1), thereby down-regulating P-gp expression. Indeed, transfection of the doxorubicin-resistant MCF-7 cells with miRNA-451 resulted in an increased sensitivity of breast cancer cells to the drug. It is tempting to speculate that cancer cells have selective mechanisms to export certain miRNAs in order to retain higher levels of P-gp necessary to shuttle chemotherapeutic drugs out.

In this context, Chen et al. [82] showed that MCF-7 cells acquired an increased survival potential through EVs released by corresponding docetaxel-resistant lines. Microarray analysis revealed once again a specific subset of miRNAs (including miRNA-222 and miRNA-452) in the “resistant EVs”. The incubation of sensitive MCF-7 cells with resistant EVs resulted in a reduction of intracellular PTEN and APC4 mRNAs known to be targeted by miRNA-222 and miRNA-452, respectively. Although protein levels had unfortunately not been analysed, the authors speculated that these oncomiRs act by down-regulating tumor suppressors. Furthermore, miRNA-21 and miRNA-155 from EVs were identified as important players in the cross-talk between neuroblastoma cells and monocytes. Co-culture experiments showed that miRNA-21 released from neuroblastoma cells led to a TLR8- and NF-кB-dependent secretion of EV-containing miRNA-155 from monocytes. Once taken up by neuroblastoma cells, miRNA-155 targeted the telomeric repeat-binding factor 1 (TERF1) inducing an increased growth when the cells were treated with cisplatin [83].

### Drug efflux pumps

Among the different reasons for drug resistance in malignancies, the up-regulation of efflux pumps such as ABC transporters is often responsible for transporting drugs out of cells [84]. Bebawy and colleagues [85] demonstrated for the first time by flow cytometry that EVs transfer functional P-glycoproteins (P-gp), well characterised ABC transporters, from drug-resistant to drug-sensitive human acute lymphoblastic leukemia cells. Corcoran et al. [86] described the potential role of EVs in transferring phenotypic changes associated with docetaxel-resistance in prostate cancer cells: an induced resistance in sensitive prostate cancer cells cultured in the presence of EVs derived from resistant cells was scored. The authors suggested P-gp to be potentially involved in the newly-acquired resistance as P-gp was expressed by both resistant prostate cells and in corresponding EVs whereas it was undetectable in the sensitive parental cells. Likewise, proteomic analysis of EVs secreted from sensitive prostate cancer cells compared to those from docetaxel-resistant cells revealed a different profile, with “resistant EVs” being once again enriched in P-gp and endophilin A [87]. The presence of these proteins was also detected in the serum of a small cohort of docetaxel-resistant patients. Hence, these EV-transported proteins were proposed as predictive biomarkers for therapeutic response or development of drug resistance [87].

The relevance of P-gp delivery through EVs in the process of transferring drug resistance was also confirmed in breast cancer cells [88]. Here, P-gp was not directly transported by EVs, but its transcription was activated by the calcium permeable channel Transient Receptor Protein Channel 5 (TrpC5) in EVs released from adriamycin-resistant breast cancer cells. Uptake of these vesicles allowed the sensitive recipient cells to acquire the TrpC5 channel, leading to increased Ca^2+^-entry and the activation of the Ca^2+^-dependent transcription factor NFATc3 (nuclear factor of activated T cells isoform c3), which in turn was responsible for increased P-gp transcription [89]. Taken together, ABC transporters carried or induced by EVs seem to play a prominent role in the development of drug resistance by providing the cell with means to rid themselves from drugs.

Several studies demonstrated the capability of EVs to confer drug resistance; however little is known about the role of EVs in inhibition of cancer cell proliferation during chemotherapy. Bovy and colleagues [90] showed that endothelial EVs taken up by breast cancer cells were able to impair growth. In response to chemotherapeutic agents, endothelial cells released EVs containing miRNA-503. The presence of this miRNA within breast cancer cells induced a reduction of their growth and invasion potential by targeting cyclins D2 and D3. Twenty-two additional up-regulated miRNAs were detected both in resistant cells and corresponding EVs in the context of breast cancer chemoresistance [91], 12 of which were significantly up-regulated in biopsies taken after neoadjuvant chemotherapy. Similar to miRNAs, long non coding RNAs (lncRNAs) have also been described to transfer drug resistance traits. The lncRNA linc-VLDLR enriched in EVs released from HCC cells was able to modulate chemotherapeutic response to sorafenib in recipient cancer cells by upregulating the ABC transporters [92]. To date, it remains to be proven whether inhibiting EV secretion might be a therapeutic option to avoid responsive cancer cells to become unresponsive.

## Limitations of the functional analysis of EVs

Although there is little doubt that EVs and their cargo can be transferred to be functionally active in recipient cells, there are several, mostly technical issues which need to be addressed to take this field one step further Standard and generally accepted procedures and protocols should be developed for:

- **Sample preparation**. EVs can be isolated by different methods (ultracentrifugation, density gradient ultracentrifugation and precipitation reagents) and there is no generally accepted procedure yet. In this context, Van Deun et al. have clearly shown that the purification method of choice will influence the purity of the vesicle population and downstream results [6]. Although it is well accepted by the community to isolate EVs through density gradient ultracentrifugation, there is an ongoing effort to find alternatives especially for complex body fluids such as plasma or urine where also the volume is a limiting factor. Moreover, density gradient ultracentrifugation is time-consuming and difficult to implement on a daily basis in clinical routine. Size exclusion chromatography, ultrafiltration and immunoprecipitation with specific antibodies have recently been tested by several groups [93–95] and might become convincing EV isolation methods in future.
- **Purity of the isolated EVs**. The essential requirements to define an EV population are: i) providing a general overview of the protein composition including proteins that should not be present in EVs, ii) performing transmission electron microscopy and nanoparticle-tracking analysis and/or flow cytometry to understand the purity of the isolates and using proper controls in functional studies.
- **Quantification**. The number of EV particles, micrograms of proteins, nanograms of EV-RNA need to be accurately determined.
- **Sensitivity of visualisation** of EV trafficking should be further improved to allow for analysis of EV functions under physiological conditions.
- **Characterisation of EV cargos** in different cellular settings with a focus on protein, miRNA and lipid profiles. The next task in this field will be to develop sensitive and specific tools to overcome the above issues. Only then will we be able to completely understand the potential of EVs as new targets in anti-neoplastic treatments and/or as new biomarkers for early detection of pathological conditions.

## Conclusions

The discovery of EVs as multi-component signaling complexes mediating intercellular communication through the delivery of molecules such as miRNAs and proteins has raised a particular interest for the use of these microvesicles as potential cancer biomarkers. Indeed, EVs present in body fluids might represent a snapshot of the status of the cancer cell at a specific time point providing highly sensitive and specific cancer markers. The simultaneous production of different subpopulations of EVs has been confirmed in many publications [96–98]. In this context, the biggest challenge the field is currently facing is the isolation and precise characterisation of the different vesicle populations and their corresponding functions. Questions such as i) different EV production and concentration in diseased versus healthy cells, ii) a clear discrimination between cancer cell-released EVs from surrounding stromal or healthy cells as well as iii) specificity of EV uptake will all have to be addressed in future studies. Only when most of these points are elucidated, can we begin to target certain subpopulation of vesicles for therapeutic purposes.

Taken together in order to exploit EVs as potential biomarkers or therapeutical targets, several technical obstacles will have to be tackled in the near future: the procedures for EV isolation and quantification need to be standardised to avoid the observed discrepancies and to dissect which other molecule classes are present in EVs and whether they get sorted into vesicles by chance or by targeted yet unknown processes. In addition, further studies on lipid composition and alteration of EVs will provide a more comprehensive understanding of their role in the biological function of EVs and also their potential impact on recipient cells. Nevertheless and in support of the above reviewed evidence, EVs can be regarded as interesting and important “homing pigeons” carrying specific messages from one place to the other. It remains to be shown how the cargo is selected and sorted and whether these processes are generally targeted or coincidental.

## Abbreviations

AP1S2, adaptor-related protein complex 1, sigma 2 subunit; APC4, anaphase promoting complex subunit 4; DLS, dynamic light scattering; EFNA3, Ephrin-A3; EGF, epidermal growth factor; eGFP, enhanced green fluorescent protein; EM, electron microscopy; EVs, extracellular vesicles; FCM, flow cytometry; FMT, fluorescence mediated tomography; HIF1a, Hypoxia-inducible factor 1-alpha; HMGA2, high-mobility group AT-hook 2; HPLC, high-performance liquid chromatography; JAK-STAT, Janus kinase, signal transducer and activator of transcription; lncRNA, long non coding RNA; M6PR, Mannose-6-Phosphate Receptor; mdr1, multidrug resistant gene 1; MHC, major histocompatibility complex; MRI, magnetic resonance imaging; MVBs, multivesicular bodies; NFATc3, nuclear factor of activated T cells isoform c3; NTA, nanoparticle tracking analysis; PET, positron emission photography; P-gp, P-glycoprotein; PI3K, Phosphoinositide 3-kinase; PTEN, phosphatase and tensin homolog; RISC, RNA-induced silencing complex; SNAREs, soluble NSF attachment protein receptor; SOCS5, Suppressor of cytokine signaling 5; TGFβ, transforming growth factor β; TrpC5, Transient Receptor Protein Channel 5; ABC transporters, ATP-binding cassette transporters; TSG101, tumor susceptibility gene; EE, early endosomes
